# Molecular Genetic Analysis of the *PLP1 *Gene in 38 Families with *PLP1*-related disorders: Identification and Functional Characterization of 11 Novel *PLP1 *Mutations

**DOI:** 10.1186/1750-1172-6-40

**Published:** 2011-06-16

**Authors:** Serena Grossi, Stefano Regis, Roberta Biancheri, Matthew Mort, Susanna Lualdi, Enrico Bertini, Graziella Uziel, Odile Boespflug-Tanguy, Alessandro Simonati, Fabio Corsolini, Ercan Demir, Valentina Marchiani, Antonio Percesepe, Franco Stanzial, Andrea Rossi, Catherine Vaurs-Barrière, David N Cooper, Mirella Filocamo

**Affiliations:** 1 S.S.D. Lab. Diagnosi Pre-Postnatale Malattie Metaboliche, IRCCS G. Gaslini, Genova, Italy; 2 U.O. Neuropsichiatria Infantile, IRCCS G. Gaslini, Genova, Italy; 3 Institute of Medical Genetics, School of Medicine, Cardiff University, Heath Park, Cardiff CF14 4XN, UK; 4 Laboratory of Molecular Medicine, Unit of Neuromuscular Disorders, Bambino Gesu' Children's Hospital, Rome, Italy; 5 Child Neurology Dept., Fondazione IRCCS Istituto Neurologico, C. Besta Milano, Italy; 6 INSERM, UMR 931, CNRS 6247, Clermont Université, GReD, Faculté de Médecine, Clermont Ferrand, France; 7 APHP, Reference Center for Rare Disease "Leukodystrophies", Service de Neurologie Pédiatrique et Maladies Métaboliques, Hôpital Robert Debré, Paris, France; 8 Department of Neurological, Neuropsychological, Morphological and Motor Sciences Section of Neurology-Child Neurology and Psychiatry Unit, Verona, Italy; 9 Gazi University, School of Medicine, Department of Child Neurology, Ankara, Turkey; 10 U.O. Neuropsichiatria Infantile Azienda, Ospedaliera S.Orsola-Malpighi, Bologna, Italy; 11 Unit of Medical Genetics, Dept. of Mother and Child, University of Modena, Modena, Italy; 12 Servizio di Consulenza Genetica, Centro Provinciale di Coordinamento della Rete delle Malattie Rare, Azienda Sanitaria dell'Alto-Adige, Bolzano, Italy; 13 Servizio di Neuroradiologia Pediatrica, IRCCS G. Gaslini, Genova, Italy

## Abstract

**Background:**

The breadth of the clinical spectrum underlying Pelizaeus-Merzbacher disease and spastic paraplegia type 2 is due to the extensive allelic heterogeneity in the X-linked *PLP1 *gene encoding myelin proteolipid protein (PLP). *PLP1 *mutations range from gene duplications of variable size found in 60-70% of patients to intragenic lesions present in 15-20% of patients.

**Methods:**

Forty-eight male patients from 38 unrelated families with a PLP1-related disorder were studied. All DNA samples were screened for *PLP1 *gene duplications using real-time PCR. *PLP1 *gene sequencing analysis was performed on patients negative for the duplication. The mutational status of all 14 potential carrier mothers of the familial *PLP1 *gene mutation was determined as well as 15/24 potential carrier mothers of the *PLP1 *duplication.

**Results and Conclusions:**

*PLP1 *gene duplications were identified in 24 of the unrelated patients whereas a variety of intragenic *PLP1 *mutations were found in the remaining 14 patients. Of the 14 different intragenic lesions, 11 were novel; these included one nonsense and 7 missense mutations, a 657-bp deletion, a microdeletion and a microduplication. The functional significance of the novel *PLP1 *missense mutations, all occurring at evolutionarily conserved residues, was analysed by the *MutPred *tool whereas their potential effect on splicing was ascertained using the *Skippy *algorithm and a neural network. Although *MutPred *predicted that all 7 novel missense mutations would be likely to be deleterious, *in silico *analysis indicated that four of them (p.Leu146Val, p.Leu159Pro, p.Thr230Ile, p.Ala247Asp) might cause exon skipping by altering exonic splicing elements. These predictions were then investigated *in vitro *for both p.Leu146Val and p.Thr230Ile by means of RNA or minigene studies and were subsequently confirmed in the case of p.Leu146Val. Peripheral neuropathy was noted in four patients harbouring intragenic mutations that altered RNA processing, but was absent from all *PLP1*-duplication patients. Unprecedentedly, family studies revealed the *de novo *occurrence of the *PLP1 *duplication at a frequency of 20%.

## Background

Mutations in the *PLP1 *gene are responsible for a wide range of X-linked white matter disorders, collectively termed *PLP1*-related disorders, which together represent a continuum of neurological features, that characterize conditions ranging from Pelizaeus-Merzbacher disease (PMD, MIM# 312080) with severe CNS involvement to the milder spastic paraplegia type 2 (SPG2, MIM# 312920) [1-3]. The PMD phenotype, which includes both the severe 'connatal' and 'classic' forms of the disease, typically manifests with nystagmus, hypotonia, cognitive impairment, severe spasticity and ataxia. In the 'connatal' form, nystagmus is present at birth and may be associated with stridor and hypotonia. Ambulation and speech are usually not achieved. The classical form of PMD is usually characterized by the onset in early infancy of nystagmus, initial hypotonia leading to spasticity, ataxia, variable extrapyramidal involvement and cognitive impairment. Supported ambulation may be achieved, although it may eventually be lost during follow-up. The so called "*PLP1 *null syndrome" is characterized by the occurrence of peripheral neuropathy that is typically absent in the other *PLP1*-related disorders. SPG2 may be either 'complicated' or 'pure' according to the presence or absence of symptoms other than spastic gait and autonomic dysfunction [3]. Brain magnetic resonance imaging (MRI) reveals a diffuse hypomyelinating leukoencephalopathy in children with the PMD phenotype, whereas in SPG2, either patchy abnormalities on T2-weighted images or a more diffuse leukoencephalopathy may occur [3].

The proteolipid protein gene (*PLP1*; MIM# 300401) contains seven exons and spans ~17 kb of chromosome Xq22.2 [4,5]. *PLP1 *encodes both the 277 amino acid proteolipid protein 1 (PLP) and its 242 amino acid isoform, DM20, which is derived from the use of a developmentally regulated internal splice donor site within *PLP1 *exon 3 [6,7]. The *in vivo *functional significance of the two distinct isoforms has not yet been fully elucidated [8,9]. PLP and DM20 are however known to be differentially expressed both during development and within different regions of the nervous system [10]. DM20, ubiquitously present both in the myelinating Schwann cells and in non-myelinating cells, is the predominant product during embryonic stages of development [11,12], but is overtaken postnatally by PLP which is abundantly expressed in oligodendrocytes and accounts for 17% of the total myelin protein [13]. The amino acid sequence of PLP has been highly conserved during mammalian evolution, with human, mouse and rat PLP sequences being completely homologous to each other [14].

The remarkable variety of mutations in the *PLP1 *gene that lead to the arrest of myelination can be grouped into two main categories: *PLP1 *gene duplications of variable size (60-70%) and intragenic sequence variants (15-20%) [2,3,15,16]. The deletion of the entire *PLP1 *gene has been reported in barely 2% of patients [17,18] whilst position effect rearrangements have been reported in a very small number of individuals with both PMD and SPG2 [19,20]. Finally, triplication and quintuplication of the *PLP1 *gene also occur, albeit very rarely [2,3]. To date, more than 160 different disease-causing *PLP1 *mutations have been reported [see the Human Gene Mutation Database [http://www.hgmd.org] [21] and the Leiden Locus-Specific Database (http://grenada.lumc.nl/LOVD2/MR/home.php)].

To date, our clinical and diagnostic center has provided a definitive diagnosis for 47 unrelated patients with PLP1-related disorders. We have previously reported the study of 9 unrelated patients, 8 of whom were males carrying the *PLP1 *gene duplication [22,23] whilst the remaining female harboured a very large duplication of Xq with random X inactivation that led to brain hypomyelination together with multiple congenital anomalies [24]. Here we report the molecular characterization of the *PLP1 *defect in a further 38 families with PLP1-related disorders, comprising a total of 43 patients. Molecular findings from this patient series have revealed that 24 of the unrelated patients possessed *PLP1 *gene duplications whereas the remaining 14 patients harboured mostly novel *PLP1 *coding sequence or splicing-relevant mutations.

## Methods

### Patients

The present patient series comprised a total of 43 male patients from 38 unrelated families with a diagnosis of a *PLP1*-related disorder. The diagnosis was predicated upon clinical evaluation and was supported by MRI studies performed in all patients except one. The main clinical and neuroradiological findings of these patients are summarized in Table 1.

**Table 1:** Clinical, neuroradiological and molecular findings of the patients

Fam/Pt ID	Age at onset	Age at last evaluation	Clinical symptoms at onset	Developmental delay/ Mental retardation	Neurological findings	Peripheral neuropathy	Clinical severity score#	Brain MRI findings	Molecular findings	
									
									Genotype	Nature of mutation ♣
**1**	Birth	8 y	Nystagmus Muscular hypotonia	Severe MR	Axial hypotonia Limb spasticity Pyramidal signs	+	0	Hypo (Supra/Infra)	c.453G>T (p.Lys151Asn)**♠**	Inherited

**2**	Birth	5 m	Nystagmus Stridor	+	Spastic tetraparesis	Absent	0	Hypo	c.552C>G (p.Cys184Trp)	Inherited

**3**	Birth	4 y	Nystagmus Muscular hypotonia	+	Muscular hypotonia	NT	0	Hypo (Supra/Infra)	c.689C>T (p.Thr230Ile)	Inherited

**4**	Birth	12 y	Nystagmus Muscular hypotonia	Severe MR	Muscular hypotonia Pyramidal signs	Absent	1	Hypo (Supra/Infra)	c.89C>A (p.Ala30Glu)	Inherited

**5**	2 m	2 y	Nystagmus	+	Spastic tetraparesis	Absent	1	Hypo (Supra/Infra)	c.505T>C (p.Cys169Arg)	Inherited

**6**	Birth	3 y	Nystagmus Muscular hypotonia	Mild MR	Muscular hypotonia Pyramidal signs	Absent*	1	Hypo	c.554_564del11 **♠ **(p.Gln185LeufsX15)	Inherited

**7**	Birth	18 y	Nystagmus Muscular hypotonia	Severe MR	Muscular hypotonia Pyramidal signs	Absent	1	Hypo	c.634T>C	Inherited

	Birth	2 y	Nystagmus Muscular hypotonia	Mild MR	Muscular hypotonia Pyramidal signs	Absent	1	Hypo	(p.Trp212Arg)	

**8**	3 m	4 y	Nystagmus Muscular hypotonia	Mild MR	Spastic tetraparesis	+	2	Hypo (Supra/Infra)	c.205C>T (p.Gln69X)**♠**	Inherited

**9**	8 m	9 y	Nystagmus Developmental delay	Mild MR	Spastic tetraparesis	+	3	Hypo	c.1-329_c.1_c.4 +324del657**♠**	Inherited

**10**	4 m	14 y	Nystagmus Muscular hypotonia	+	Spastic tetraparesis	+	3	Hypo (Supra/Infra)	c.134_140dup7 (p.Ile47IlefsX4)**♠**	Inherited

**11**	6 m	30 y	Nystagmus	-	Limb spasticity Poor tendon reflexes	NT	4	NT	c.436C>G (p.Leu146Val)**♠**	Inherited

**12**	Birth	11 m	Nystagmus Stridor	+	Muscular hypotonia	NT	♦	Hypo	c.83G>T (p.Gly28Val)	Inherited

**13**	Birth	11 m	Nystagmus	Mild MR	Muscular hypotonia	Absent	♦	Hypo (Supra/Infra)	c.476T>C (p.Leu159Pro)	*De novo*

**14**	1 m	3 m	Nystagmus Muscular hypotonia	+	Muscular hypotonia	NT	♦	Hypo	c.740C>A (p.Ala247Asp)	Inherited

**15**	Birth	5 y	Nystagmus Muscular hypotonia	Severe MR	Muscular hypotonia Pyramidal signs	Absent	0	Hypo (Supra/Infra)	*PLP1*dup	NA

**16**	Birth	18 m	Nystagmus Muscular hypotonia Stridor	Mild MR	Muscular hypotonia	Absent	0	Hypo (Supra/Infra)	*PLP1*dup	*De novo*

**17**	1 m	17 y	Nystagmus	Mild MR	Muscular hypotonia	Absent	1	Hypo (Supra/Infra)	*PLP1*dup	NA

**18**	3 m	10 y	Nystagmus Muscular hypotonia	+	Muscular hypotonia Pyramidal signs Dystonia Ataxia	Absent	1	Hypo	*PLP1*dup	Inherited

	2 m	11 y	Nystagmus Muscular hypotonia	Moderate MR	Spastic tetraparesis Dystonia	Absent	1	Hypo		

**19**	1 m	2 y	Nystagmus	Moderate MR	Muscular hypotonia Pyramidal signs Dystonia	Absent	1	Hypo (Supra/Infra)	*PLP1*dup	Inherited

**20**	1 m	4 y	Nystagmus	Mild MR	Muscular hypotonia Pyramidal signs	Absent	2	Hypo	*PLP1*dup	Inherited

**21**	2 m	4 y	Nystagmus	+	Muscular hypotonia	Absent	2	Hypo	*PLP1*dup	NA

**22**	2 m	4 m	Nystagmus Muscular hypotonia	Mild MR	Muscular hypotonia	Absent	2	Hypo	*PLP1*dup	NA

**23**	4 m	2 y	Nystagmus	Mild MR	Muscular hypotonia	Absent	2	Hypo	*PLP1*dup	Inherited

**24**	Birth	22 y	Nystagmus	Severe MR	Spastic tetraparesis Dystonia	Absent	2	Hypo	*PLP1*dup	Inherited

**25**	8 m	2 y	Nystagmus Developmental delay	+	Spastic tetraparesis	Absent	2	Hypo	*PLP1*dup	Inherited

**26**	1 m	4 y	Nystagmus	Severe MR	Pyramidal signs Dystonia	Absent	2	Hypo	*PLP1*dup	Inherited

**27**	5 m	2 y	Nystagmus	Severe MR	Muscular hypotonia	Absent	2	Hypo	*PLP1*dup	*De novo*

	15 d	5 y	Nystagmus Seizures	Moderate MR	Spastic tetraparesis Dystonia	Absent	2	Hypo		

**28**	1 m	8 y	Nystagmus	+	Pyramidal signs Dystonia	Absent	2	Hypo	*PLP1*dup	Inherited

	4 m	29 y	Nystagmus Seizures	+	Pyramidal signs Dystonia	Absent	3	Hypo		

**29**	1 y	25 y	Nystagmus Developmental delay	Severe MR	Spastic tetraparesis	Absent	3	Hypo	*PLP1*dup	Inherited

	1 y	23 y	Nystagmus Developmental delay	Severe MR	Spastic tetraparesis	Absent	3	Hypo		

**30**	3 m	5 y	Nystagmus	Mild MR	Muscular hypotonia Pyramidal signs	Absent	3	Hypo (Supra/Infra)	*PLP1*dup	Inherited

**31**	Birth	3 m	Nystagmus Muscular hypotonia	Mild MR	Muscular hypotonia	Absent	3	Hypo	*PLP1*dup	NA

**32**	2 m	6 y	Nystagmus	Moderate MR	Pyramidal signs Ataxia	Absent	3	Hypo	*PLP1*dup	Inherited

**33**	6 y	30 y	Nystagmus Learning difficulties Behavioural problems	Moderate MR	Limb spasticity Ataxia	Absent	4	Hypo	*PLP1*dup	NA

**34**	3 m	11 m	Nystagmus Muscular hypotonia	+	Muscular hypotonia Pyramidal signs	Absent	♦	Hypo (Supra/Infra)	*PLP1*dup	Inherited

**35**	1 m	7 m	Nystagmus	+	Muscular hypotonia	Absent	♦	Hypo	*PLP1*dup	NA

**36**	3 m	11 m	Nystagmus Muscular hypotonia	+	Muscular hypotonia Dystonia	Absent	♦	Hypo	*PLP1*dup	NA

**37**	1 m	11m	Nystagmus	+	Muscular hypotonia	Absent	♦	Hypo	*PLP1*dup	*De novo*

**38**	3 m	8 m	Nystagmus Muscular hypotonia	+	Muscular hypotonia	Absent	♦	Hypo (Supra/Infra)	*PLP1*dup	NA

Legend: Fam = Family; Pt = Patient; d = day(s) m = month(s); y = year(s); NT = not tested; * indicates peripheral neuropathy present in the symptomatic mother; MR = mental retardation; MRI = magnetic resonance imaging; Hypo = hypomyelination; Supra = supratentorial; Infra = infratentorial; # according to Cailloux et al. [35]; ♦indicates score not assessable in patients below 12 months of age; NA = not available; **♠**flags mutations predicted to alter RNA processing; ♣indicates data based on molecular findings in the mother's DNA samples

### Ethical aspects

Following ethical guidelines, all cell and nucleic acid samples were obtained for analysis and storage with the patients' (and/or a family member's) written informed consent. The consent was sought using a form approved by the local Ethics Committee.

### Cell culture

Fibroblast and lymphoblast cells were cultured according to standard procedures. The cell lines were cultured and maintained in RPMI medium (EuroClone, Gibco, Paisley, UK) containing 15% FCS and penicillin/streptomycin, in a humidified atmosphere containing 5% CO_2 _at 37°C.

Oli-neu (murine oligodendrocyte precursor) cells (kindly provided by Dr. J. Trotter, University of Mainz, Germany) were cultured in Sato medium containing 1% horse serum [25].

### Molecular analysis

All DNA samples were initially screened for the presence of the *PLP1 *gene duplication using real-time PCR. In a subsequent step, sequencing analysis was performed on every patient who was found to be negative with respect to the gene duplication. Where necessary, further investigations on RNA samples were carried out whenever possible. Genomic DNA was extracted from peripheral blood leukocytes and/or cultured cell lines using standard methods or suitable kits [QIAmp DNA blood mini kit (Qiagen Inc., Valencia, CA, USA) or the Nucleon BACC3 kit for blood and cell cultures (Amersham Biosciences, Bucks, UK)].

Total RNA was extracted from patient fibroblasts using an RNeasy mini plus kit (Qiagen, Valencia, CA, USA) and reverse transcribed by means of an Advantage RT-for-PCR kit (BD Biosciences Clontech, Mountain View, CA, USA).

#### PLP1 gene dosage determination by real-time PCR

DNA samples from the patients were assessed for *PLP1 *gene dosage by real-time PCR using amplicon *PLP1*gen, located in exon 3 of the *PLP1 *gene, and amplicon GAPgen, located in exon 7 of the glyceraldehyde-3-phosphate dehydrogenase (*GAPDH*) gene which was used as a reference sequence [26].

*PLP1 *gene dosage was determined by real-time PCR amplification from genomic DNA. Amplicons defined by two primers and a TaqMan probe were designed for the real-time PCR runs using the Primer Express 1.5 software (Applied Biosystems, Foster City, CA, USA) according to previously reported requirements [26]. The real-time PCR experiments were performed using the Applied Biosystems 7500 Real-Time PCR System with TaqMan chemistry as previously described [26]. The standard curve method, with amplification of the target and reference sequences in separate tubes, was employed (User Bulletin #2. Relative quantitation of gene expression; http://www.appliedbiosystems.com/). Samples were run in quadruplicate whereas standards were run in duplicate. All TaqMan probes used were labelled with FAM as the reporter fluorophore at their 5'-end and with BHQ1 as the quencher at their 3'-end. Primers and probes were purchased by TIB Molbiol (Genoa, Italy).

#### PLP1 Mutation Analysis

All *PLP1 *gene exons and exon-intron boundaries were PCR amplified to yield 5 amplicons using 5 sets of primers designed by reference to the *PLP1 *genomic sequence (GenBank-EMBL Accession No. NC_000023.10). Reverse transcript-PCR (RT-PCR) was performed using sets of primers designed by reference to the *PLP1 *mRNA sequence (GenBank accession No. NM_000533.3) Primers and PCR reaction conditions are given in Additional file 1, Table S1a. All amplicons were purified and directly sequenced using an ABI 377 DNA automated sequencer with dye terminator cycle sequencing kits (Applied Biosystems, Foster City, CA, USA).

Putative mutations were confirmed by sequencing duplicate PCR products or by digesting PCR products with a specific restriction endonuclease whose recognition site was consequently altered. If the mutation neither created nor destroyed a restriction site, amplification was carried out using PCR-mediated site-directed mutagenesis that served to introduce a new restriction enzyme cleavage site [27]. The possibility that the novel mutations were polymorphisms was excluded by determining that none of 50 female healthy control subjects (100 alleles) carried any of these alterations. Further, by definition, none of the novel variants had ever been reported before (as either a disease-causing mutation or a polymorphism) in any previous study.

#### Bioinformatic analysis of PLP1 variants

Missense variants in the *PLP1 *gene were analysed by means of a computational model, *MutPred *[28,29]. *MutPred *was designed to model the effect of observed changes in structural and functional sites within a protein between wild-type and mutant protein sequences. *MutPred *can also be used to generate hypotheses as to the underlying molecular mechanism(s) responsible for disease pathogenesis for any given mutation. The effects of coding region variants upon splicing [splice site disruption, cryptic splice site activation and exon skipping, as a consequence either of the loss of an exonic splicing enhancer (ESE) and/or the gain of an exonic splicing silencer (ESS)] were ascertained using *Skippy *[30] and a neural network for splice site prediction [31]. The NI-ESR hexamers [32] (979 ESEs and 496 ESSs) formed the basis of the ESE and ESS motifs used in this analysis, as this set had previously been identified as providing the strongest signal for identifying exon skipping variants [30].

#### Mutation c.436C>G (p.Leu146Val): minigene splicing construct and quantitative real-time RT-PCR-based evaluation of PLP1 and DM20 transcript content

A PCR product containing portions of exon/intron 2, exon/intron 3 and exon 4 was obtained from a healthy control using primers PP2F and PP4R that introduced *Cla*I and *Eco*RI sites (Additional file 1, Table S1b). The product was cleaved with *Cla*I and *Eco*RI and cloned into a similarly cleaved pcDNA3.1/V5-His-TOPO/LacZ vector (Invitrogen, San Diego, CA). A recombinant in-frame LacZ-PLP1-LacZ minigene, containing the genomic region between exons 2 and 4 of the *PLP1 *gene, was then obtained. The cloned fragment was sequenced, thereby confirming its identity to the *PLP1 *gene reference sequence. Subsequently, the c.436C>G (p.Leu146Val) mutation was introduced into the vector using the QuikChange II Site-Directed Mutagenesis Kit (Stratagene Agilent, Santa Clara, California, USA) according to the manufacturer's instructions. Wild-type and mutant plasmid constructs were transfected into murine oligodendroglial Oli-neu cells using the lipofectamine 2000 Transfection Reagent (Invitrogen, San Diego, CA) according to the manufacturer's instructions. Transfected cells were harvested 72 hrs after transfection, and RNA was extracted using an RNeasy Plus Mini kit (Qiagen, Valencia, CA, USA) and reverse transcribed using the Advantage RT-for-PCR kit (BD Biosciences Clontech, Mountain View, CA, USA). Reverse transcription was performed using a LacZ reverse primer (LACT2R) in order to avoid retrotranscription of endogenous *Plp1 *gene transcripts from the Oli-neu cells. RT-PCR was performed using the primers reported in Additional file 1, Table S1b.

To evaluate *PLP *and *DM20 *transcript content quantitatively in cells transfected with wild-type and mutant constructs, a real-time PCR analysis was performed, as previously described, using a *DM20 *transcript-specific amplicon (D2), a *PLP *transcript-specific amplicon (P2), both encompassing exons 3 and 4, and a (*DM20*+*PLP*) transcript-specific amplicon (P23B) encompassing exons 2 and 3 (Additional file 1, Table S1c). While amplicons D2 and P2 were used as targets, amplicon P23B was used as a reference. Using this experimental approach, we were able to derive the *DM20*/(*DM20*+*PLP*) and *PLP*/(*DM20*+*PLP*) ratios for cells transfected with wild-type and mutant constructs. Wild-type and mutant cDNAs were run in quadruplicate, each well containing the cDNA obtained from 10 ng RNA. A plasmid containing the *DM20 *cDNA was used to generate the standard curve for the D2 amplicon whilst a plasmid containing the *PLP *cDNA was used to generate the standard curves for the P2 and the P23B amplicons, respectively. Standards were run in duplicate. Standard wells contained 10-2, 10-3, 10-4, 10-5, 10-6, 10-7 ng/μl plasmid DNA.

### Mutation nomenclature

All mutations are described according to the recommended nomenclature [33,34]. Nucleotide numbering was derived from the human *PLP1 *cDNA sequence (GenBank-EMBL Accession No. NM_000533.3) ascribing the A of the first ATG translational initiation codon as nucleotide +1. Amino acid residue numbering was as derived for the human PLP protein (GenBank-EMBL Accession No. NP_000524.3).

## Results and Discussion

In this study we report the molecular genetic analysis of 43 patients with *PLP1*-related disorders from 38 unrelated families. The clinical, neuroradiological and molecular findings obtained in relation to the 43 patients are summarized in Table 1.

### Clinical aspects

As reported in Table 1, 15 patients (pts) (from 14 families) were found to possess *PLP1 *mutations whereas the remaining 28 patients (from 24 families) harboured a *PLP1 *duplication. Age at onset of clinical symptoms ranged from birth to 1 year old, with the exception of one patient (pt #33) who was reported as having nystagmus, learning difficulties and behavioural problems in childhood. The age at last evaluation ranged from 3 months to 30 years (Table 1). Nystagmus, either isolated or associated with other symptoms, was the presenting symptom in all patients. The other presenting symptoms in the 43 patients were: seizures (2/43), stridor (3/43), developmental delay (4/43) and muscular hypotonia (18/43). Developmental delay and mental retardation (ranging from mild to severe) were reported during follow-up in all patients except for pt #11. Neurological findings revealed abnormal muscular tone with prevailingly axial hypotonia or diffuse muscular hypotonia in the youngest subjects associated with pyramidal signs in 11/43. Increased muscle tone, either limb spasticity (3 pts) or tetraparesis (11 pts), was evident in older patients. Dystonia was present in 9 patients (in association with pyramidal signs in 8/9) and ataxia in three patients. Peripheral neuropathy (confirmed by nerve conduction velocity studies) was present in four subjects with *PLP1 *mutations but was absent in all patients harbouring a *PLP1 *duplication. A reduced amplitude of the compound muscle action potential, consistent with an axonal neuropathy, was identified in two subjects (pts #1 and #9), whereas a mixed axonal and demyelinating process was diagnosed in two further patients (pts #8 and #10).

Clinical severity, assessed according to the score proposed by Cailloux et al. [35], ranged from 0 to 4 on the basis of the maximal level of motor acquisition. Three out of five patients with the lowest score (i.e. no postural achievement) belonged to the *PLP1 *mutation group, whereas the remaining two patients harboured *PLP1 *duplications. Nine patients, having achieved head control, had a score of 1: of these, five were in the *PLP1 *mutation group whereas four were in the *PLP1 *duplication group. Eleven patients, 10 of whom carried a *PLP1 *duplication, were able to sit without assistance, thereby obtaining a score of 2. Of the 8 patients who acquired the ability to walk with support (viz. score 3), two were in the *PLP1 *mutation group whereas six were in the *PLP1 *duplication group. Finally, only two patients, one with a *PLP1 *mutation and the other with a *PLP1 *duplication, acquired the ability to walk without support, thereby obtaining a score of 4. Brain MRI indicated hypomyelination in all 42 patients who had undergone brain MRI. In 14/17 patients, from whom more detailed neuroradiological data were available, infratentorial myelin deficiency was present in addition to the supratentorial hypomyelination that was evident in all patients.

### Molecular studies

**Figure 1:**
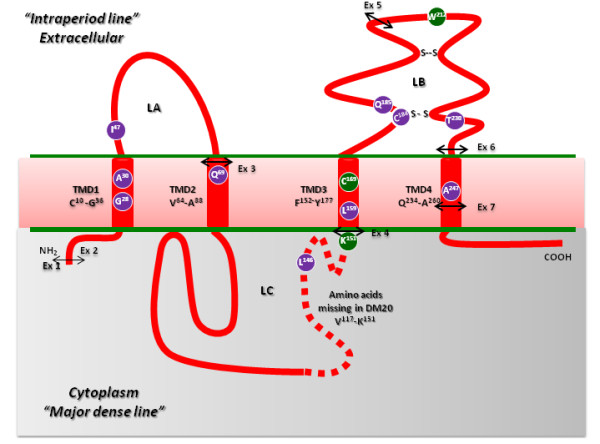
Distribution of *PLP1 *gene mutations identified in our series of patients with PLP1-related disorders in relation to the proposed model* of the tetra-span proteolipid proteins, PLP and DM20. With respect to the missense mutations reported here, the amino acid residues affected by novel mutations are shown in purple, whereas residues harbouring previously reported mutations are given in green. The four predicted transmembrane domains (TMD1-4) are depicted, the first and last amino acids being indicated for each TMD. The PLP1-specific region (which is absent from DM20) is denoted by a dotted line. The locations of the two disulfide bridges (Cys184-Cys228 and Cys201-Cys220) within loop B (LB) are marked "S--S". The double arrow indicates the relative placement of the exon-exon (Ex) junctions superimposed upon the PLP1 protein. *model according to Popot et al, [36] and Weimbs and Stoffel [37]

The laboratory diagnostic protocol included an initial screening, by real-time PCR, of all DNA samples for the presence of the *PLP1 *gene duplication. In the next step, patients negative for the gene duplication underwent *PLP1 *sequencing analysis. Analysis of the present patient series revealed a *PLP1 *gene duplication in 24 of the unrelated patients whereas a variety of intragenic *PLP1 *mutations was found to underlie disease pathogenesis in the remaining 14 unrelated patients (Table 1). Table 2 reports the characteristics of the 14 different genomic lesions identified in these patients as a result of sequencing the exons and exon-intron boundaries of the *PLP1 *gene. Figure 1 depicts the distribution of the coding sequence mutations in relation to the proposed model of the tetra-span proteolipid proteins, PLP and DM20 [36,37].

**Table 2:** Characteristics of *PLP1 *gene mutations identified in 14 unrelated patients and MutPred analysis of the missense mutations

Location	cDNA mutation*	Mutation Effect	MutPred analysis of missense mutations						Refer ence
				
			Probability of deleterious mutation		No. of ESE binding sites loses	No. of ESS binding sites gained	Skippy Log Odds Ratio (LOR) Total	Splice site disruption prediction	
**5'UTR-Ex 1-Intr 1**	**c.1-329_c.1_c.4 +324del657**	**r.?**	.		.	.	.		Present study

	**c.134_140dup7**	**p.Ile47IlefsX4**	.		.	.	.		Present study

**Exon 2**	**c.83G>T**	**p.Gly28Val**	0.80	SS Loss of loop (P = 0.0252)	0	1	0.052		Present study

	**c.89C>A**	**p.Ala30Glu**	0.84		0	0	-5.884		Present study

	**c.205C>T**	**p.Gln69X**	.		0	2	1.290		Present study

	**c.436C>G**	**p.Leu146Val**	0.67		0	5	0.356		Present study

**Exon 3**	c.453G>T	p.Lys151Asn	0.63		0	0	-1.284	*[predicted to abolish 5'SS with Neural Network] already reported in the cited ref*	Hobson et al. [38]

	**c.476T>C**	**p.Leu159Pro**	0.87	SS Helix > Sheet (P = 0.0266), Gain of glycosylation at T160 (P = 0.0342)	6	0	1.360		Present study

**Exon 4**	c.505T>C	p.Cys169Arg	0.91		4	0	1.360		Mimault et al. [48]

	**c.552C>G**	**p.Cys184Trp**	0.88		2	0	-0.929		Present study

	**c.554_564del11**	**p.Gln185LeufsX15**	.						Present study

**Exon 5**	c.634T>C	p.Trp212Arg	0.79	Loss of catalytic residue at L210 (P = 0.0114), Gain of methylation at W212 (P = 0.0245)	0	0	-1.284		Cailloux et al. [35]

	**c.689C>T**	**p.Thr230Ile**	0.68		5	1	2.696		Present study

**Exon 6**	**c.740C>A**	**p.Ala247Asp**	0.88		1	2	2.006		Present study

* Nucleotide numbers are derived from cDNA *PLP1 *sequence (GenBank-EMBL accession no. NM_000533.3) taking as nucleotide +1 the A of the first ATG translation initiation codon; Bold type denotes novel mutation; Ex = exon; Intr = Intron; ESE = exonic splicing enhancers; ESS = exonic splicing silencers; *Skippy *Log Odds Ratio (LOR) score is the output from the *Skippy *tool [30], the *Skippy *LOR score represents the likelihood that the combination of ESR changes consequent to a substitution are associated with exon skipping. The higher the *Skippy *LOR score, the more likely the combination of ESR changes will be associated with an exon skipping event. The prediction of splice site disruption was evaluated using a neural network. The column 'Location' refers to the exon within which the mutation occurs and also the exon that could potentially be skipped due to ESE loss and/or ESS gain.

Eleven of the mutations were novel and included (i) 7 missense mutations (p.Gly28Val, p.Ala30Glu, p.Leu146Val, p.Leu159Pro, p.Cys184Trp, p.Thr230Ile, p.Ala247Asp) and one nonsense mutation (p.Gln69X); (ii) a large deletion (c.1-329_c.1_c.4+324del657) involving the removal of 657-bp from the untranslated, exonic and intronic regions surrounding exon 1; (iii) a microdeletion (c.554_564del11) which results in a frameshift (p.Gln185LeufsX15) that is predicted to introduce a premature stop codon 15 residues downstream; and (iv) a microduplication in exon 2 (c.134_140dup7) which would also be predicted to lead to a frameshift (p.Ile47IlefsX4) with premature truncation of the protein four amino acids downstream.

The question of the pathological authenticity of the novel *PLP1 *sequence alterations detected was addressed by (i) searching dbSNP (http://www.ncbi.nlm.nih.gov/SNP) for their annotation and excluding their presence, (ii) screening 50 healthy female control subjects (100 alleles) for each alteration and determining that none carried any of these alterations, (iii) analysing the evolutionary conservation of the amino acid residues affected, (iv) employing the *MutPred *program [28,29] to assess each missense mutation, and (v) using *Skippy *[30] and a neural network for splice site prediction [31] to explore the potential for mutational consequences for splicing.

### Analysis of the evolutionary conservation of amino acid residues affected by missense mutations

Support for the pathological relevance of the missense mutations identified in the present study, came from the analysis of the extent of evolutionary conservation of the mutated residues in 9 orthologous (vertebrate) PLP proteins. Computational analysis (http://www.ensembl.org/) revealed that all missense mutations occurred at evolutionarily conserved amino acid residues. Moreover, seven of the novel mutations affected amino acid residues (namely Gly28, Ala30, Leu159, Cys184, Trp212, Thr230 and Ala247) that were invariant even when zebrafish PLP was considered (Additional file 2, Table S2).

### *MutPred *analysis

In an attempt to assess the functional relevance or otherwise of the novel *PLP1 *missense mutations identified, we employed the *in silico *analysis tool, *MutPred *[28,29]. *MutPred *predicted that all 7 novel missense mutations listed in Table 2 would be deleterious (*MutPred *general score > 65) whilst confident *in silico *hypotheses for the underlying mechanism of pathogenesis were generated for two of them. Thus, protein secondary structure was predicted to be altered by the missense mutations p.Gly28Val (P = 0.025) and p.Leu159Pro (Helix to Sheet; P = 0.027); the latter replacement (p.Leu159Pro) was also predicted to give rise to a gain of glycosylation at p.Thr160 (P = 0.034).

The effect of the coding region variants upon splicing [including splice site disruption, cryptic splice site activation and exon skipping via loss of exonic splicing enhancers (ESE), and/or gain of exonic splicing silencers (ESS)] was ascertained using *Skippy *[30] and a neural network for splice site prediction [31]. Whereas this latter algorithm predicted that only p.Lys151Asn would abolish the 5' splice site, as already reported by Hobson et al. [38], the results of the *Skippy *analysis (Table 2) indicated that four other missense mutations (p.Leu146Val, p.Leu159Pro, p.Thr230Ile, p.Ala247Asp) could potentially cause exon skipping by altering exonic splicing elements (ESR). In all four instances, the exon predicted to be skipped would be the same as that harbouring the mutation (Table 2, Location). The dramatic copy number changes of ESE or ESS motifs for p.Leu146Val and p.Thr230Ile further identified these novel missense mutations as being high confidence 'exon skipping' candidates suitable for further *in vitro *analysis. Indeed, c.436C>G (p.Leu146Val) appears to lead to a net gain of 5 exon splicing silencer (***ESS***) motifs, whilst c.689C>T (p.Thr230Ile) results in the net loss of 5 exon splicing enhancers (ESE) as well as the gain of an ESS motif. The potential impact of both p.Leu146Val and p.Thr230Ile on *PLP1 *mRNA processing was therefore investigated by means of RNA or minigene studies.

### Deduced consequences of *PLP1 *mutations for mRNA processing

The postulated effect of mutations c.436C>G (p.Leu146Val) and c.689C>T (p.Thr230Ile) on *PLP1 *mRNA processing was explored *in vitro *as follows:

#### Mutation c.436C>G (p.Leu146Val)

**Figure 2:**
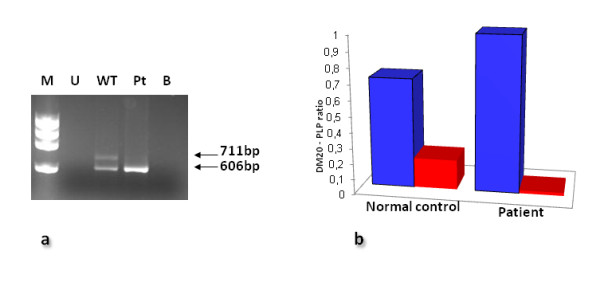
**Mutation c.436C>G (p.Leu146Val)**. **a) **RT-PCR performed with the minigene-specific 31GF/LACT2R primers (Additional file 1, Table S1b) on cDNA transfected into Oli-neu cells. Whereas the expected *PLP1 *and *DM20 *products were present in cells transfected with the wild-type construct (WT), only the *DM20 *product was present in cells transfected with the patient mutant construct (Pt). No product was present in untransfected cells (U) or in the negative control lacking DNA (B). ΦX174 DNA *Hae*III digest used as a marker (M). **b) **Real-time PCR quantification using [*DM20*]- [*PLP*]- and [*DM20+PLP*]-specific amplicons confirmed the altered ratio of *DM20*/(*DM20*+*PLP*) (blue bars) and *PLP*/(*DM20*+*PLP*) (red bars) in the patient carrying the mutation Leu146Val as compared to the healthy control.

Since no RNA sample was available from pt #11, the functional consequences of the mutation c.436C>G (p.Leu146Val) were assessed using a recombinant minigene construct containing a *PLP1 *exon 2-exon 4 fragment, cloned into an expression vector. Oli-neu cells were then transfected with the wild-type (c.436C) and mutant (c.436G) versions of the construct, and RNA samples extracted. To avoid the synthesis of endogenous murine Oli-neu *Plp1 *transcripts, first strand cDNA was reverse transcribed using a minigene-specific primer. RT-PCR products from the mutant construct (c.436G) revealed only the presence of the DM20-specific transcript (the PLP-specific transcript being totally absent) when compared with the wild-type construct (c.436C) (Figure 2a). To further corroborate these data, we evaluated the [*DM20*]/[*DM20+PLP*] and [*PLP*]/[*DM20*+*PLP*] ratios in cells transfected with the wild-type and mutant constructs, by real-time PCR quantification using [*DM20*]- [*PLP*]- and [*DM20+PLP*]-specific amplicons. As expected, whereas the [*DM20*]/[*DM20*+*PLP*] ratio displayed by the mutant construct was 0.998 (compared to 0.707 for the wild-type control), the [*PLP*]/[*DM20*+*PLP*] ratio associated with the mutant construct was 0.019 vs 0.192 for the wild-type construct (Figure 2b). These findings indicate that the mutation c.436C>G (p.Leu146Val) abolished *PLP *isoform-specific splicing, thereby confirming our *in silico *predictions. Thus, only the transcript corresponding to the DM20 isoform was found in association with the p.Leu146Val *PLP1 *mutation.

#### Mutation c.689C>T (p.Thr230Ile)

Based on the *in silico *prediction (loss of 5 ESE motifs and gain of 1 ESS motif), an alteration of the splicing phenotype was expected for this missense mutation. RT-PCR analysis of *PLP1 *RNA isolated from the fibroblasts of pt #3 (using the PLPF-PLP1R primer set reported in Additional file 1, Table S1c), revealed instead the presence only of normally-sized 1040-bp and 915-bp products of the *PLP *and *DM20 *isoforms, respectively, both of which harboured the missense mutation.

### Characterization of the gross deletion (c.1-329_c.1_c.4 +324del657)

The genomic breakpoints of the large genomic deletion were found to overlap 4 nucleotides of exon 1 (including the start codon ATG), the flanking upstream 329-bp untranslated region (5'UTR) and the downstream 324-bp intronic regions, respectively. We then evaluated its consequences for RNA processing by means of quantitative real-time PCR. These experiments, performed on RNA extracted from the fibroblasts of pt #9, indicated that the PLP transcript isoform was completely absent, whereas the DM20 transcript isoform was present at a reduced level, ~50% as compared to healthy controls (data not shown).

The recombinational mechanism underlying the gross deletion was then explored. Since no significant homologies were detected at the deletion breakpoints, we infer that non-homologous end-joining (NHEJ) may have been responsible for this deletion [39,40]. The underlying mechanism may also have involved sequence elements present at the 5' breakpoint [i.e. an A-rich sequence and a polypurine tract (both 17 bp long)] and 3' breakpoint [a tract highly homologous to the translin site consensus sequence], previously reported as being over-represented at translocation breakpoints [41].

### Genotype-phenotype correlations

A remarkable number of different *PLP1 *mutations have been reported as being responsible for disease pathogenesis in PLP1-related disorders. It has generally been observed that patients with *PLP1 *gene duplications display a range of clinical severity, independent of the extent of the duplication [22,42]. This variation in clinical severity is also evident in our patient series; indeed, the clinical severity scores varied between 0 and 4 in different patients.

Peripheral neuropathy, never reported in patients with *PLP1 *gene duplications, has been frequently found to be associated with intragenic *PLP1 *mutations that either abrogate *PLP1 *expression (null alleles), affect the *PLP*-specific region or alter certain *PLP1 *splice sites [43,44]. In accordance with these general observations, peripheral neuropathy was absent in all 24 families with a *PLP1 *duplication (Table 1). Interestingly, only those patients (#1, #8, #9, #10) who harboured *PLP1 *mutations that were predicted to result in the absence of the PLP isoform, exhibited peripheral neuropathy [as confirmed by nerve conduction velocity studies (NCVs)]. Although NCVs in another patient (#6) who harboured a null allele were within the normal range for his age (2 years), his carrier mother exhibited spastic paraplegia and peripheral neuropathy with severe slowing of nerve conduction velocity.

Analysing the clinical severity score of each patient in the *PLP1 *mutation group in relation to the type of mutation, the most severe phenotype (score = 0) (Table 1 pts #1, #2 and #3) was found to be associated with (i) the p.Lys151Asn mutation, which occurred at the alternative 5' splice donor site of the *PLP *and *DM20 *isoforms, and which altered levels of both the *PLP- *and *DM20-*encoding RNAs [38]; (ii) the novel substitution (p.Cys184Trp), that replaced a cysteine known to be involved in disulfide bridge formation (Cys184-Cys228) (Figure 1) with a tryptophan (Trp), and which is likely to destabilize the folded protein and induce the UPR (unfolded protein response) pathway leading to apoptosis [45]. Recently, the Cys184Arg substitution has been reported as a cause of the 'classic' form of PMD [46] thereby supporting our contention that any defect involving the disulfide bridge (Cys184-Cys228) would strongly impair correct PLP folding; (iii) the substitution of an amino acid residue (p.Thr203Ile) that is common to both the PLP and DM20 isoforms and is evolutionarily invariant even when zebrafish PLP is considered (Additional file 2, Table S2).

The least severe phenotype (clinical severity score = 4) was noted in patient #11 who harboured the lesion c.436C>G (p.Leu146Val); *in vitro *studies confirmed that this mutation results in altered *PLP1 *RNA processing, leading to the production of the *DM20 *isoform in the absence of the PLP isoform (Figure 2). The findings outlined above are therefore in accord with previous observations of patients with *PLP1*-related disorders in whom a relatively mild syndrome is associated with mutations that affect the PLP isoform but not the DM20 isoform [2,3,16].

### Family studies

The mutation analysis was extended wherever possible to all female family members. So far, although *de novo *mutations have been reported in several instances of *PLP1 *point mutations, *de novo *mutation appears at the very least to be much rarer (and is perhaps unprecedented) in the case of *PLP1 *duplications (see Hodes et al. [47], and Mimault et al. [48], for discussion of this still contentious issue). Mimault et al. [48] analysed the maternal mutation status of 56 families; whereas in 22 families with *PLP1 *gene mutations, 32% of mothers were found not to be carriers of the mutation concerned, these authors found that only three (9%) of 34 mothers of *PLP1 *duplication patients (analyzed by a multiplex endpoint PCR-based approach) were not carriers. On the basis of a statistical analysis which revealed a significant male duplication imbalance, these authors suggested that the *PLP1 *duplications could have arisen in the grandpaternal germline.

In our own patient series, we analyzed all 14 obligate carrier mothers of the familial *PLP1 *gene mutation and 15/24 obligate carrier mothers of the *PLP1 *duplication. In contrast to the previously reported results, the *PLP1 *mutations were all found to be maternally inherited with the sole exception of pt #13, in whom the missense mutation appears to have occurred *de novo *(7%). However, the *PLP1 *duplication was found to have occurred *de novo *in 3 of the 15 mothers analyzed (20%) (Table 1). The proportion of *PLP1 *duplications occurring *de novo *in our series is significantly higher than the 9% reported by Mimault et al. [48] The limited number of cases analyzed here (15 mothers) notwithstanding, the *de novo *occurrence of the *PLP1 *gene duplication at a frequency of 20% is quite unprecedented. If these novel and unexpected findings are confirmed on a larger number of patients, the previously postulated high prevalence of a grandpaternal origin for the *PLP1 *duplication will have to be re-evaluated. The relatively high frequency of *de novo PLP1 *duplication reported here would certainly concur with recent findings indicating a rather higher *de novo *mutation rate for CNVs than for point mutations [49,50]. It may well be that the discrepancy between our own data and those previously reported is due to the greatly improved analytical methods currently in use for the identification of the *PLP1 *duplication.

Although a high rate of *de novo *duplication was documented in our study, gonadal mosaicism could not be formally excluded in the patient's mothers who were found to be negative with respect to a specific mutation. Therefore, in clinical practice, prenatal testing is recommended for those couples potentially at risk. Female carriers of *PLP1*-related disorders are generally normal neurologically, but may manifest mild to moderate signs of late onset disease [3]. It is however known that those mutant *PLP1 *alleles that are responsible for hemizygous males being affected with comparatively mild neurological disease symptoms, may also give rise to neurological manifestations in female heterozygotes [51]. In addition, owing to favourably skewed X inactivation in heterozygous females with a *PLP1 *duplication, the risk of a carrier female being clinically affected is lowest in the case of a *PLP1 *duplication and highest in the presence of a *PLP1 *null allele [52]. None of the 15 mothers studied here who were heterozygous for the *PLP1 *duplication were clinically affected. On the other hand, of the 14 mothers who were carriers of an intragenic *PLP1 *mutation, neurological impairment (spastic paraplegia and peripheral neuropathy) was reported only in the mother (Table 1, Fam #6) who harboured a putative null allele (p.Gln185LeufsX15).

## Competing interests

The authors are not aware of any financial or non-financial competing interests related to this manuscript.

## Authors' contributions

SR, SG, and CVB performed the molecular genetic studies and the sequence alignment, and helped to draft the manuscript. SL assisted with the molecular studies. FC performed and managed cell line cultures and biobanking. MM carried out the bioinformatics analysis. RB, EB, GU, OBT, AS, ED, VM, AP, FS contributed to the acquisition, organization and analysis of the clinical data. AR contributed to the evaluation of neuroradiological data. RB coordinated the clinical data and helped to draft the manuscript. MF coordinated the study and drafted the manuscript with the assistance of DNC who also made a substantial contribution to placing the findings in their broader scientific context. All authors read and approved the final manuscript.
